# Detection and Characterisation of Colistin-Resistant *Escherichia coli* in Broiler Meats

**DOI:** 10.3390/microorganisms12122535

**Published:** 2024-12-09

**Authors:** Abu Zubayer Tanzin, Chandan Nath, Md. Raihan Khan Nayem, Md Abu Sayeed, Shahneaz Ali Khan, Ricardo Soares Magalhaes, John I. Alawneh, Mohammad Mahmudul Hassan

**Affiliations:** 1 Remount Veterinary and Farm Corps, Bangladesh Army, Savar, Dhaka 1341, Bangladesh; tanjin04@gmail.com (A.Z.T.); raihannayem@gmail.com (M.R.K.N.); 2 Faculty of Veterinary Medicine, Chattogram Veterinary and Animal Sciences University, Chattogram 4225, Bangladesh; chandannath227@gmail.com (C.N.); shahneaz@cvasu.ac.bd (S.A.K.); 3 National Centre for Epidemiology and Population Health (NCEPH), College of Health and Medicine, The Australian National University, Canberra, ACT 2601, Australia; 4 Queensland Alliance for One Health Sciences, School of Veterinary Science, The University of Queensland, Gatton, QLD 4343, Australia; r.magalhaes@uq.edu.au; 5 Plant Biosecurity and Product Integrity, Biosecurity Queensland, Department of Agriculture and Fisheries, Brisbane, QLD 4000, Australia; john.alawneh@daf.qld.gov.au

**Keywords:** LBMs, supermarket, meat, colistin-resistant *E. coli*, multidrug resistance, mobilised colistin resistance gene

## Abstract

The irrational use of antimicrobials has led to the emergence of resistance, impacting not only pathogenic bacteria but also commensal bacteria. Resistance against colistin, a last-resort antibiotic, mediated by globally disseminated plasmid-borne mobile colistin resistance (*mcr*) genes, has raised significant global concerns. This cross-sectional study aimed to investigate the antimicrobial resistance patterns of colistin-resistant *Escherichia coli* (*E. coli*) and mobilised colistin resistance (*mcr* 1–5) genes from broiler meat. A total of 570 broiler samples (285 liver and 285 muscle) were collected from 7 supermarkets and 11 live bird markets (LBMs) in Chattogram metropolitan areas of Bangladesh. The isolation and identification of *E. coli* were carried out using standard bacteriological and molecular techniques. Antimicrobial susceptibility testing (AST) was performed using the Kirby–Bauer disc diffusion method, and colistin’s minimum inhibitory concentration (MIC) was determined by the broth microdilution (BMD) method. Colistin-resistant isolates were further tested for the presence of *mcr* (1–5) genes using polymerase chain reaction (PCR). Out of the 570 samples, 311 (54.56%; 95% confidence interval: 50.46–58.60) were positive for *E. coli*. AST results showed the highest resistance to sulphamethoxazole–trimethoprim (89.39%), while the highest susceptibility was observed for cefalexin (62.70%). A total of 296 isolates (95.18%) were found to be multidrug-resistant (MDR), with the multiple antibiotic resistance (MAR) index ranging from 0.38 to 1. Additionally, 41 isolates (13.18%) exhibited resistance to five antimicrobial classes, with resistance patterns of CIP + SXT + AMP + DO + TE + CT. A total of 233 isolates (74.92%) were resistant to colistin (MIC > 2 mg/L). A strong correlation between colistin resistance and the presence of the *mcr*-1 gene was observed (r = 1). All phenotypic colistin-resistant *E. coli* isolates carried the *mcr*-1 gene, while no isolates were positive for *mcr* (2–5). The detection of *mcr* genes in *E. coli* strains from poultry sources poses a significant risk, as these resistance genes can be transferred to humans through the food chain. The prevalence of multidrug-resistant *Escherichia coli* and the *mcr*-1 gene in poultry products in Bangladesh presents a significant public health and food safety concern.

## 1. Introduction

Antimicrobial resistance (AMR), a critical global health challenge, has intensified with the indiscriminate use of antibiotics, especially in livestock production, where poultry farming alone accounts for 60% of global antibiotic consumption. This alarming trend risks propelling the world towards a “pre-antibiotic era”, in which previously treatable infections and minor injuries could become life-threatening due to the absence of effective therapies [1,2,3]. In 2015, the World Health Organization (WHO) launched the Global Action Plan (GAP) to address these issues. This plan promotes ‘One Health’ to tackle AMR by understanding the interdependence of human, animal, and environmental health [1]. The GAP seeks to combat antimicrobial resistance (AMR) in developing countries like Bangladesh by enhancing awareness, strengthening surveillance and research, reducing infection rates, optimising antibiotic use, and ensuring sustainable funding to address AMR effectively [4]. In Bangladesh, due to agricultural expansion and growing populations, the overall demand for animal protein, mainly poultry, is predicted to rise in the upcoming years [5]. Poultry industries largely contribute to economic growth and employment opportunities in this country [6]. However, this industry’s reliance on antibiotics for growth promotion and infection control has contributed to a concerning increase in AMR [2]. A number of factors are associated with the elevated risk of AMR, and the sale of antimicrobials as over-the-counter medicine has worsened the situation [7]. The country’s diverse health system, which employs unqualified informal health providers, is complicating this problem day by day. This, along with unapproved pharmaceutical marketing, has led to the misuse of antimicrobials [8]. This enormous, unregulated market in Bangladesh is difficult to govern due to insufficient human, technical, and logistical capabilities [9]. To promote the rational use of antibiotics and curb the spread of AMR, Bangladesh is working to adopt a WHO GAP-compliant AMR containment National Action Plan (NAP) [10]. Therefore, we need detailed data on the current AMR situation across the human, animal, and environmental sectors to coordinate and effectively implement the NAP. Food animal production systems in Bangladesh employ antibiotics extensively to boost output and prevent infectious disease outbreaks [7,11]. Although antibiotics may boost growth, treat diseases, and avoid illness in livestock [8], the abuse of these antibiotics in food animals, especially in intensive farming systems, has increased AMR in foodborne pathogens and other commensal species. Among the different commensal species, *E. coli*, a ubiquitous bacterium commonly found in the intestines of human and warm-blooded animal species, has been widely studied as an AMR model organism [12]. Antibiotics used to treat diseases can promote *E. coli*’s environmental adaptability by fostering resistance, making it an ideal organism for the study of AMR. Recently, multidrug-resistant (MDR) *E. coli* has become more common in poultry [9,10] in contrast to in other food animal species [10]. The use of antibiotics in poultry can lead to the emergence of resistance in bacteria found in animals, which can then be transmitted to humans via consumption or direct contact. Furthermore, antibiotics may accumulate in animal muscle, and these residues can result in adverse health outcomes in humans, including toxicity, allergic reactions, and the emergence of resistance [13]. Resistance against colistin, one of the last-resort antibiotics used to treat complicated multidrug-resistant (MDR), Gram-negative bacterial infections, is a growing concern. The development of colistin resistance has been associated with the plasmid-mediated mobile colistin resistance gene (*mcr*-1) [14,15,16]. Additionally, the identification of the *mcr*-2 gene, which shares 76.7% nucleotide similarity with *mcr*-1, in *E. coli* strains from Belgian isolates indicates how *E. coli* adapts to its environment [17,18]. Overall, the coexistence of the *mcr*-1 gene with other resistance genes like *bla*_NDM_ and *tet* A in different *E. coli* strains may form pandrug-resistant superbugs [19]. Hence, this resistance mechanism must be contained, because *Enterobacterales* strains with *mcr*-1 can spread internationally through trade and travel [20]. Limited studies have examined multidrug and colistin resistance in *E. coli* species isolated from poultry species in Bangladesh [21,22,23,24,25,26,27]. An earlier study reported that about 53.75% of *E. coli* and 50% of *Salmonella* species from Bangladeshi commercial poultry farms were colistin-resistant [28]. Thus, it is crucial to determine the multidrug resistance patterns of colistin-resistant *E. coli* due to the risk of transmission of poultry-origin *E. coli* through the food chain in Bangladesh. Therefore, this study investigates the prevalence of colistin-resistant *E. coli* in meat isolated from different sources along with the MDR patterns of colistin-resistant *E. coli*.

## 2. Materials and Methods

### 2.1. Study Area and Sampling

We conducted this study between 15 January and 30 July 2021 in the Chattogram Metropolitan Area (CMA) of the Chattogram district (district—a small administrative area), the second-largest city and main port of Bangladesh. We randomly selected eleven live bird markets (LBMs) and seven supermarkets to collect broiler muscle and liver samples. From each LBM, we collected 10 liver and 10 muscle samples, and from each supermarket, we collected 25 liver and 25 muscle samples. A total of 570 samples were collected, comprising 285 breast muscle and 285 liver samples from broilers (Supplementary Table S1). After collection, the samples were immediately transported in a cool box to the Department of Physiology, Biochemistry, and Pharmacology (DPBP) laboratory at Chattogram Veterinary and Animal Sciences University (CVASU). In the laboratory, we aseptically cut the samples into small pieces, placed them in falcon tubes containing buffered peptone water (BPW) (Oxoid^TM^ Ltd., Basingstoke, UK) at a 1:9 ratio, and stored them at 4 °C until culturing.

### 2.2. Phenotypic Isolation and Identification of E. coli

Firstly, the buffered peptone water (BPW) (Oxoid^TM^ Ltd., Basingstoke, UK) broth containing samples was incubated at 37 °C overnight for pre-enrichment. After incubation, a loopful of broth (~10 µL) was inoculated onto MacConkey agar (Oxoid^TM^ Ltd., Basingstoke, UK) and incubated at 37 °C for 24 h. Large, pink-coloured colonies on the MacConkey agar plates were suspected to be *E. coli*. These colonies were subsequently inoculated onto eosin methylene blue (EMB) agar (Oxoid^TM^ Ltd., Basingstoke, UK) and incubated for 24 h at 37 °C for biochemical confirmation. The characteristic metallic sheen colony was sub-cultured onto blood agar (Oxoid^TM^ Ltd., Basingstoke, UK) and incubated at 37 °C for 24 h. Then, the colonies were preserved in Eppendorf tubes containing 700 µL brain–heart infusion (BHI) (Oxoid^TM^ Ltd., Basingstoke, UK) broth with 300 µL 15% glycerol for each isolate and stored at −80 °C for further investigation.

### 2.3. Molecular Detection of E. coli

We extracted the genomic DNA using the crude boiling method [29]. All phenotypically positive isolates were subjected to molecular identification through species-specific multiplex PCR, using primers targeting the *uid*A gene and the flanking region of the *usp*A gene (Supplementary Table S2). We present all the oligonucleotide primer sequences in Supplementary Table S1. We performed the PCR amplifications in a thermal cycler (DLAB Scientific Inc., Walnut, CA, USA) in a final reaction volume of 25 µL. The reaction mixture contained 12.5 µL of OneTaq Quick-load 2× MM with Standard Buffer (New EngladBiolabs., Ipswich, MA, USA), 0.5 µL each of forward and reverse primers (10 pmol), 1 µL of template DNA, and the required volume of nuclease-free water. The cycling conditions for the detection of *E. coli* were as follows: an initial denaturation at 94 °C for 5 min, followed by 35 cycles of denaturation at 94 °C for 10 s, annealing at 55.2 °C for 10 s, extension at 72 °C for 1 min, and a final extension at 72 °C for 10 min [30]. To visualise the PCR products, 1.5% (*w*/*v*) agarose gel was prepared using agarose powder (MP Biomedicals, Irvine, CA, USA) and 1× TAE buffer (Thermo Fisher Scientific Inc., Waltham, MA, USA). Electrophoresis was performed at 110 volts and 80 mA for 30 min. The gel was visualised using a gel documentation system (UVP UVsolo touch, Analytik Jena AG, Thermo Fisher Scientific, USA) and stained with ethidium bromide (Sigma-Aldrich, St. Louis, MO, USA). The *E. coli* ATCC 25922 was used as a positive control and nuclease-free water as a negative control.

### 2.4. Antimicrobial Susceptibility Test (AST)

We performed testing on all *E. coli* (n = 311) isolates for antimicrobial susceptibility using the Kirby–Bauer disc diffusion method [31] against a panel of seven antimicrobials from six different drug classes, each with significant public health implications and each frequently used in the poultry industry for treatment and prevention purposes. The antimicrobials used in this study were as follows: tetracycline—tetracycline (TE, 30 µg) and doxycycline (DO, 30 µg); aminoglycosides—gentamicin (CN, 10 µg); fluoroquinolones—ciprofloxacin (CIP, 5 µg); sulphonamides—sulphamethoxazole–trimethoprim (SXT, 23.75 µg + 1.25 µg); cephalosporin—cephalexin (CL, 30 µg); and penicillin—ampicillin (AMP, 10 µg) (Oxoid Ltd., UK). First, sub-culturing of the *E. coli* strain was performed in a blood agar plate incubated overnight at 37 °C. After incubation, a 0.5 McFarland inoculum of each strain was made by dissolving 2–3 colonies from the blood agar plate in a sterile glass tube containing 3 mL 0.9% NaCl saline solution. The turbidity of the inoculum was adjusted to the 0.5 McFarland standard. The inoculum was streaked evenly in three planes onto the surface of the Mueller–Hinton agar (MHA) (HiMedia, Mumbai, India) plate using a sterile cotton-tipped swab. After that, the inoculum was permitted to dry for 5 min, and the discs were placed on the agar with flamed forceps. The plates were incubated overnight at 37 °C. After incubation, the zone diameters were measured (in mm) and interpreted according to the Clinical and Laboratory Standards Institute (CLSI) guidelines [32]. *Escherichia coli* ATCC 25922 was used as a quality-control strain. Isolates showing resistance to at least three antimicrobial agents were classified as MDR [33]. The multiple antibiotic resistance (MAR) index was calculated using the method described in [34].

### 2.5. Minimum Inhibitory Concentration (MIC) of Colistin

We performed testing on all *E. coli* isolates to obtain the MIC of colistin sulphate using the broth microdilution (BMD) method, following the ISO 20776-2 standard [35]. Briefly, the preserved *E. coli* isolates were sub-cultured on blood agar (Oxoid Ltd., UK) and incubated overnight at 37 °C. Cation-adjusted Mueller–Hinton Broth (MHB) II (Sigma-Aldrich, St. Louis, MO, USA), colistin sulphate (Sigma-Aldrich, St. Louis, MO, USA), and 96-well plates were used for MIC determination. First, 50 µL of MHB II was added to each well of the microtiter plate, and 50 µL of colistin sulphate solution (512 µg/mL) was added to column 11. Then, two-fold serial dilutions were performed in reverse order from column 11 to column 2. Next, 50 µL of the 0.5 McFarland standard inoculum, prepared earlier, was added to columns 1 to 11, and the plate was incubated at 37 °C for 18–20 h. The interpretation was performed by observing the concentration of the antibiotic in the last well, where there was no visible bacterial growth. Column 1 served as the culture control (no antibiotic → bacterial growth present), and column 12 was the negative control (no antibiotic and no culture → clear fluid, no growth). *E. coli* strains NCTC 13846 (*mcr*-1-positive) and ATCC 25922 (pan-susceptible *E. coli*) were used as colistin-resistant and -susceptible controls, respectively. The MIC value was interpreted following CLSI guidelines for colistin sulphate [32].

### 2.6. Detection of mcr (1–5) Genes in Phenotypic Colistin-Resistant E. coli

We performed testing on all the phenotypic colistin-resistant *E. coli* isolates to detect *mcr* (1–5) genes with multiplex PCR, using a list of oligonucleotide primer sequences (Supplementary Table S1) and following standard procedure [36,37]. The cycling conditions were as follows: initial denaturation at 94 °C for 15 min, followed by 25 cycles of denaturation at 94 °C for 30 s, annealing at 58 °C for 90 s, and extension at 72 °C for 60 s, with a final extension at 72 °C for 10 min and holding at 4 °C. The following strains were used as positive controls for the respective *mcr* genes: *E. coli* NCTC 13846 for *mcr*-1, *E. coli* KP37 for *mcr*-2 [38], *E. coli* 2013-SQ3525 for *mcr*-3 [37], laboratory-generated *E. coli* DH5α for *mcr*-4 [39], and *Salmonella* 13-SA01718 for *mcr*-5 [40]. Additionally, the *E. coli* ATCC 25922 strain was used as a negative control [41].

### 2.7. Statistical Analysis

We entered, sorted, and cleaned all the data in Microsoft Excel 2013, and then performed descriptive analysis and calculated the 95% confidence interval (CI) using STATA-11 software. We calculated the correlation coefficient between antimicrobials and resistance genes in R software (version 4.4.1; https://www.r-project.org/, accessed on 5 September 2024) and visualised the results using the ggplot2 (ggcorrplot version 0.1.4.1) package. Heatmaps and other diagrams were constructed using GraphPad Prism 7.0 (La Jolla, CA, USA).

## 3. Results

### 3.1. Prevalence of E. coli in Different Sources and Types

Among the 570 samples we tested, a total of 311 (54.56%; 95% CI: 50.46–58.60) were positive for *E. coli*. We found that the prevalence of *E. coli* in LBMs was 59.09%, with 47.27% in liver samples and 70.91% in muscle samples. Similarly, in supermarkets, the prevalence was 51.71%, with 63.43% in liver samples and 40.0% in muscle samples (Table 1).

### 3.2. AMR Profiling with Resistant Gene Detection in E. coli Isolates

#### 3.2.1. Phenotypic AMR Profiles

**Figure 1:**
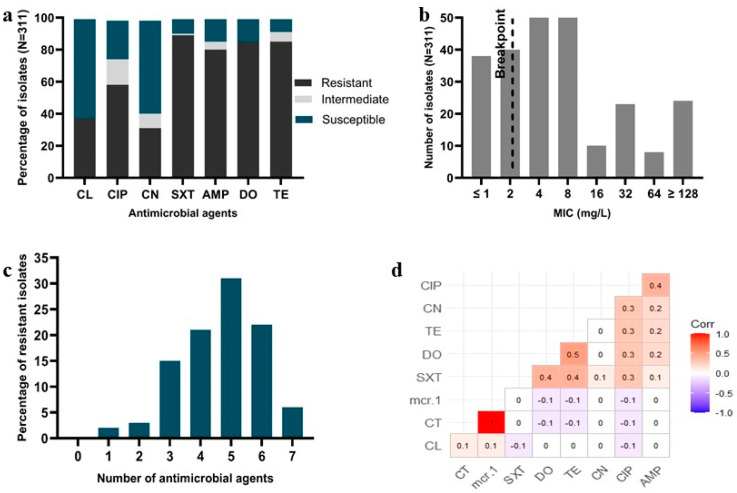
AMR profiles of *E. coli* isolated from muscle and liver samples of broiler chicken. (**a**) AMR profiles, (**b**) MIC of colistin sulphate, (**c**) MDR profiles, and (**d**) correlation coefficient of antimicrobials and resistance genes. Here, TE = tetracycline; DO = doxycycline; CN = gentamicin; CIP = ciprofloxacin; SXT = sulphamethoxazole–trimethoprim; CL = cephalexin; AMP = ampicillin; and CT = colistin.

Antimicrobial susceptibility testing (AST) revealed that *E. coli* isolates exhibited the highest resistance to sulphamethoxazole–trimethoprim (89.39%), followed by doxycycline (85.53%), tetracycline (85.21%), and ampicillin (80.71%). Conversely, cefalexin demonstrated the highest susceptibility, with 62.70% of the isolates being susceptible to it (Figure 1a).

#### 3.2.2. MIC of Colistin Sulphate

We determined that 233 isolates (74.92%) were resistant to colistin (MIC ≥ 4 µg/mL), while 78 isolates (25.08%) were susceptible (MIC ≤ 2 µg/mL). Additionally, 92 isolates (29.58%) had an MIC value of 4 µg/mL, and 8 isolates exhibited an MIC value of 64 µg/mL. The MIC values for colistin sulphate are shown in Figure 1b.

#### 3.2.3. Phenotypic MDR Patterns and Detection of *mcr* (1–5) Genes

Among the *E. coli* isolates, we identified 296 (95.18%) as MDR, with 30.54% showing resistance to five different antimicrobial classes. Notably, 5.79% of the isolates were resistant to all antimicrobials tested in this study (Figure 1c). Based on phenotypic antimicrobial resistance patterns, the highest number of MDR isolates, 41 (13.18%), exhibited resistance to five antimicrobial classes, explicitly following the CIP + SXT + AMP + DO + TE + CT resistance pattern. The correlation analysis revealed a positive correlation between CT and the *mcr*-1 gene (r = 1), between TE and DO (r = 0.5), and between AMP and CIP (r = 0.4) (Figure 1d). All the phenotypic colistin-resistant *E. coli* isolates (233, 74.92%) carried the *mcr*-1 gene, with no other *mcr* genes detected.

#### 3.2.4. AMR Profile and Its Correlation with *E. coli* Isolates

**Figure 2:**
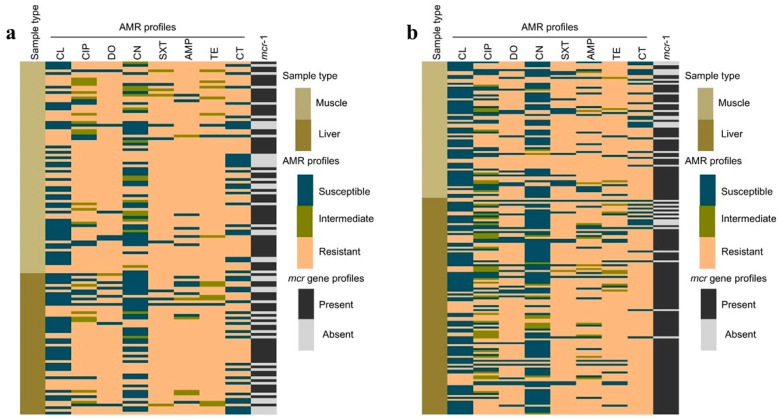
Source-specific AMR profiles of different samples, showing AMR patterns across liver and muscle samples—(**a**) LBM; (**b**) supermarket.

We estimate that the multiple antibiotic resistance (MAR) index of the MDR isolates ranged from 0.38 to 1, suggesting that the *E. coli* isolates were exposed to multiple antibiotics, which is indicative of contamination during or after the meat processing stage. Meat processing environments often involve handling, equipment, and water sources that can be potential reservoirs of antimicrobial-resistant bacteria, contributing to cross-contamination (Table 2). We also illustrate the source-specific AMR profiles for *E. coli* isolates from different sample types in Figure 2. The heatmaps compare AMR profiles from liver and muscle samples collected from LBMs and supermarkets, highlighting varying levels of resistance to antimicrobials such as cefalexin, ciprofloxacin, doxycycline, gentamicin, sulphamethoxazole–trimethoprim, ampicillin, tetracycline, and colistin, along with the presence of the *mcr*-1 gene responsible for colistin resistance (Figure 2).

## 4. Discussion

AMR refers to a bacterium’s ability to withstand antibiotic effects. Bacteria can develop this resistance through natural mechanisms, genetic mutations, or resistance acquired from other species. AMR genes may encode intrinsic resistance that exists or develops through spontaneous mutation or evolutionary change after the antimicrobial’s emergence [42]. Many countries cannot treat *E. coli* infection because it has become fluoroquinolone-resistant [43]. In many parts of the world, this treatment is currently ineffective for more than half of patients. Moreover, colistin, a last-resort antibiotic for treating life-threatening infections caused by carbapenem-resistant *Enterobacterales* like *E. coli*, is increasingly encountering resistance, with several unresolved questions surrounding the mechanisms and drivers of this resistance [44]. AMR has become an emerging issue in developing countries like Bangladesh. This study examined the spread of the *mcr*-1 to *mcr*-5 genes in the gut bacteria of Bangladeshi chickens, specifically targeting *E. coli* from live bird markets and supermarkets. In this study, the prevalence of *E. coli* was 54.56%, which is comparable to the findings of previous studies conducted in Bangladesh, reporting prevalence rates of 58% [45] and 61.67% [46], respectively. *E. coli* was detected in 57.19% in liver samples and51.93% in muscle samples. These findings align with [47], which reported a prevalence of 54.40% in muscle samples and 56% in liver samples. The reported prevalence of *E. coli* in live bird markets and supermarkets was 59.09% and 51.17%, respectively, which is consistent with [47], which reported that the prevalence of *E. coli* in both live bird markets and supermarkets was 53.33% and 63.33%, respectively. The antimicrobial susceptibility test showed that sulphamethoxazole–trimethoprim was the most resistant (89.07%). It was followed by tetracycline (85.85%), ampicillin (80.71%), ciprofloxacin (58.84%), doxycycline (50.16%), cephalexin (34.73%), gentamicin (30.87%), and colistin (20.58%). Previous studies reported a high resistance to ampicillin, tetracycline, and sulphamethoxazole–trimethoprim of *E. coli* isolated from poultry [48,49,50]. Earlier studies support our findings that around 42% and 29% of isolates were susceptible to colistin and gentamycin, respectively [51,52,53]. Despite the lack of consistent statistics on its usage, polymyxin E (colistin), a last-resort antibiotic for *Enterobacterales* infections, is widely used in poultry practices in Bangladesh [54]. In line with the controversy surrounding the use of antimicrobial agents in livestock and poultry production, evidence suggests that antibiotic usage plays an essential role in the emergence of AMR bacteria [55]. MDR microorganisms withstand at least three antimicrobial classes representing various antimicrobials [33]. This high emergence of MDR in the study area may be due to the extensive use of antimicrobials in poultry and humans [55]. Researchers found different amounts of MDR *E. coli* in different sources: for example, in 75% of foods sold on the street [56], in 62.9% of drinking water [53], and in 100% of poultry [49]. The findings suggest that MDR strains could lead to “superbugs” that are immune to medication. Genotypically, liver and muscle isolates exhibit a higher concentration of resistance genes than other tissues. In this study, we identified the *mcr*-1 gene in 27.02% of colistin-resistant isolates, a finding that aligns with the study conducted in [57]. In muscle samples, the prevalence of the *mcr*-1 resistance gene was 25%, and in liver samples, it was 29.41%. In live markets and supermarkets, the prevalence of *mcr*-1 is 28.57% and 25%, respectively, which is higher than in an earlier study [58], where 5.3% of colistin-resistant *E. coli* isolates had the *mcr*-1 gene. However, over time, the prevalence of *mcr*-1 and *mcr*-3 rose to as high as 30% and 8.3%, respectively, in China [58]. The *mcr*-1 prevalence in all *E. coli* isolates tested was 3.2% (10 out of 311). This is similar to the prevalence reported in [59], which detected the *mcr* gene in 1.6% of *E. coli* isolates obtained from cattle in Spain. The *E. coli* isolates from poultry farms predominantly carried the *mcr*-1 gene in agreement with findings reported in [60]. The present study suggests that irrational antibiotic usage in chicken farms may cause commensal bacteria like *E. coli* to develop resistance. Therefore, a rational approach needs to be developed to curb the misuse of antimicrobials at the government level. This study has significant value because AMR threatens the effectiveness of life-saving treatments, making infections harder to treat and increasing mortality rates in both animals and humans. It directly impacts public health by escalating healthcare costs, prolonging hospital stays, and increasing the risk of untreatable infections spreading within communities. Effectively managing AMR in poultry requires the judicious use of antibiotics to mitigate the risks identified in this study. Developing policies and practices that promote responsible antibiotic use in agriculture demands a science-driven approach and a deeper understanding of AMR dynamics.

## 5. Conclusions

This study revealed a high prevalence of *E. coli* isolates in liver and muscle samples from broiler chickens, with notable differences in contamination levels between live bird markets (LBMs) and supermarkets. A large proportion of these isolates were MDR, particularly resistant to antimicrobials such as sulphamethoxazole–trimethoprim, tetracycline, and ampicillin. Furthermore, we found the *mcr*-1 gene, which encodes colistin resistance, in all phenotypic colistin-resistant isolates, though the frequency of its presence varied between sample and market types. The plasmid-borne nature of *mcr*-1 in commensal *E. coli* facilitates its potential transmission to other bacterial hosts and enables the co-transfer of additional antibiotic resistance genes (ARGs), increasing the risk of “superbugs” that are MDR and pose significant treatment challenges in humans, particularly through the food chain, including poultry. AMR in poultry not only exacerbates the challenge of treating bacterial infections in animals but also facilitates the spread of difficult-to-treat strains to humans. While this does not directly increase AMR in humans, it contributes to infections with MDR pathogens, leading to higher morbidity and mortality rates. This, in turn, perpetuates cycles of poverty and human suffering by increasing the burden of disease in affected communities. To mitigate this, the prudent and rational use of antimicrobials in poultry farms is crucial. Implementation of strict policies, mass awareness programs, and training for poultry handlers is essential to ensure the responsible use of antibiotics to prevent the spread of AMRs across the community, which will maintain the potency of crucial antimicrobials and avoid the emergence of superbugs in human and animal health.

## Figures and Tables

**Table 1:** An overview of the samples collected, the frequency of *E. coli*, and colistin resistance status across different sources and sample types.

Source	Sample Type	No. of *E. coli, n* (%)	Phenotypic Resistance to Colistin Sulphate, n (%)	No. of *mcr*-1, n (%)
LBMs	Liver (n = 110)	52 (47.27)	32 (61.54)	32 (100)
	Muscle (n = 110)	78 (70.91)	52 (66.67)	52 (100)
	Subtotal (n = 220)	130 (59.09)	84 (64.62)	84 (100)
Supermarket	Liver (175)	111(63.43)	98 (88.29)	98 (100)
	Muscle (n = 175)	70 (40)	51 (72.86)	51 (100)
	Subtotal (n = 350)	181 (51.71)	149 (82.32)	149 (100)
Total (N = 570)		311 (54.56)	233 (74.92)	233 (100)

**Table 2:** Phenotypic antimicrobial resistance patterns of *E. coli* isolates. Here, TE = tetracycline; DO = doxycycline; CN = gentamicin; CIP = ciprofloxacin; SXT = sulphamethoxazole–trimethoprim; CL = cephalexin; AMP = ampicillin; and CT = colistin. R = resistant; MDR = multidrug-resistant; MAR = multiple antibiotic resistance.

No of Isolates	%	Resistance Type	Phenotypic Resistance Patterns	MAR Index
41	13.18	MDR	Five classes CIP, SXT, AMP, DO, TE, CT	0.75
30	9.65	MDR	Six classes CIP, CN, SXT, AMP, DO, TE, CT	0.88
26	8.36	MDR	Six classes CL, CIP, SXT, AMP, DO, TE, CT	0.88
19	6.11	MDR	Five classes CIP, CN, SXT, AMP, DO, TE	0.75
19	6.11	MDR	Four classes SXT, AMP, DO, TE, CT	0.63
18	5.79	MDR	Seven classes CL, CIP, CN, SXT, AMP, DO, TE, CT	1
14	4.50	MDR	Three classes SXT, DO, TE, CT	0.5
12	3.86	MDR	Four classes CIP, SXT, AMP, DO, TE	0.63
11	3.54	MDR	Five classes CL, SXT, AMP, DO, TE, CT	0.75
9	2.89	MDR	Three classes SXT, AMP, DO, TE	0.5
8	2.57	MDR	Four classes CL, SXT, DO, TE, CT	0.63
8	2.57	MDR	Six classes CL, CIP, CN, SXT, AMP, DO, TE	0.88
6	1.93	MDR	Five classes CL, CIP, SXT, AMP, DO, TE	0.75
5	1.61	MDR	Six classes CL, CN, SXT, AMP, DO, CT	0.75
4	1.28	MDR	Three classes CL, AMP, CT	0.38
4	1.28	MDR	Four classes CL, AMP, DO, TE, CT	0.63
4	1.28	MDR	Five classes CN, SXT, AMP, DO, TE, CT	0.75
3	0.96	MDR	Four classes SXT, AMP, TE, CT	0.5
3	0.96	MDR	Four classes CL, CN, AMP, CT	0.5
3	0.96	MDR	Five classes CIP, SXT, DO, TE, CT	0.63
3	0.96	MDR	Four classes SXT, AMP, DO, CT	0.5
3	0.96	MDR	Five classes CIP, CN, SXT, AMP, CT	0.63
3	0.96	MDR	Five classes CIP, SXT, AMP, TE, CT	0.63
3	0.96	R	One class CT	0.13
2	0.64	MDR	Three classes AMP, TE, CT	0.38
2	0.64	MDR	Four classes CIP, CN, SXT, DO, TE	0.63
2	0.64	MDR	Five classes CL, CIP, SXT, DO, TE, CT	0.75
2	0.64	MDR	Five classes CL, CN, SXT, TE, CT	0.63
2	0.64	MDR	Three classes CIP, SXT, DO, TE	0.5
2	0.64	MDR	Three classes CIP, SXT, CT	0.38
2	0.64	MDR	Three classes CL, AMP, DO, TE	0.5
2	0.64	R	Three classes AMP, DO, CT	0.38
2	0.64	MDR	Three classes CN, AMP, CT	0.38
2	0.64	MDR	Three classes CL, SXT, CT	0.38
2	0.64	MDR	Four classes CN, SXT, AMP, DO, CT	0.63
2	0.64	MDR	Five classes CL, CN, SXT, AMP, DO, TE	0.75
1	0.32	R	One class AMP	0.13
1	0.32	R	One class SXT	0.13
1	0.32	R	Two classes DO, CT	0.25
1	0.32	R	Two classes SXT, DO, TE	0.38
1	0.32	R	Two classes AMP, DO, TE	0.38
1	0.32	R	Two classes SXT, TE	0.25
1	0.32	R	Two classes CL, DO, TE	0.38
1	0.32	R	Two classes SXT, AMP	0.25
1	0.32	R	Two classes CN, TE	0.25
1	0.32	R	Two classes SXT, CT	0.25
1	0.32	MDR	Three classes CL, SXT, DO	0.38
1	0.32	MDR	Three classes CIP, CN, CT	0.38
1	0.32	MDR	Three classes AMP, DO, TE, CT	0.5
1	0.32	MDR	Three classes CL, AMP, DO	0.38
1	0.32	MDR	Three classes CL, SXT, DO, TE	0.5
1	0.32	MDR	Three classes SXT, AMP, DO	0.38
1	0.32	MDR	Three classes SXT, DO, CT	0.38
1	0.32	MDR	Three classes CN, TE, CT	0.38
1	0.32	MDR	Four classes CL, SXT, DO, CT	0.5
1	0.32	MDR	Four classes CIP, SXT, AMP, TE	0.5
1	0.32	MDR	Four classes CIP, SXT, AMP, DO	0.5
1	0.32	MDR	Four classes CL, CIP, DO, TE, CT	0.63
1	0.32	MDR	Four classes CL, SXT, AMP, DO, TE	0.63
1	0.32	MDR	Four classes CL, CN, SXT, DO	0.5
1	0.32	MDR	Four classes CL, CIP, SXT, DO, TE	0.63
1	0.32	MDR	Four classes CL, SXT, AMP, CT	0.5
1	0.32	MDR	Four classes CIP, SXT, AMP, CT	0.5
1	0.32	MDR	Five classes CL, CN, SXT, DO, TE, CT	0.75

## Data Availability

The original contributions presented in the study are included in the article/supplementary material, further inquiries can be directed to the corresponding authors.

## Supplementary Materials

The following supporting information can be downloaded at: https://www.mdpi.com/article/10.3390/microorganisms12122535/s1, Supplementary Table S1: List of samples collected from different LBMs and supermarkets; Supplementary Table S2: Oligonucleotide primer sequences used in this study.
