# Supplementary material for: Detection and Characterisation of Colistin-Resistant Escherichia coli in Broiler Meats
Source: Microorganisms. 2024 Dec 9;12(12):2535. doi: 10.3390/microorganisms12122535 (PMC11676989; doi:10.3390/microorganisms12122535)
Supplement: Supplementary file 1 [file microorganisms-12-02535-s001.zip › Supplementary Table S1.docx]

Supplementary Table S1: List of sample collected from different LBMs and supermarkets (LBM= Live Bird Market, SM= Supermarket)

| Sources | Category | Sample type | |
| --- | --- | --- | --- |
|  |  | Liver | Muscle |
| LBMs | LBM-1 | 10 | 10 |
|  | LBM-2 | 10 | 10 |
|  | LBM-3 | 10 | 10 |
|  | LBM-4 | 10 | 10 |
|  | LBM-5 | 10 | 10 |
|  | LBM-6 | 10 | 10 |
|  | LBM-7 | 10 | 10 |
|  | LBM-8 | 10 | 10 |
|  | LBM-9 | 10 | 10 |
|  | LBM-10 | 10 | 10 |
|  | LBM-11 | 10 | 10 |
| Supermarkets | SM-1 | 25 | 25 |
|  | SM-2 | 25 | 25 |
|  | SM-3 | 25 | 25 |
|  | SM-4 | 25 | 25 |
|  | SM-5 | 25 | 25 |
|  | SM-6 | 25 | 25 |
|  | SM-7 | 25 | 25 |
