# Supplementary material for: Detection and Characterisation of Colistin-Resistant Escherichia coli in Broiler Meats
Source: Microorganisms. 2024 Dec 9;12(12):2535. doi: 10.3390/microorganisms12122535 (PMC11676989; doi:10.3390/microorganisms12122535)
Supplement: Supplementary file 1 [file microorganisms-12-02535-s001.zip › Supplementary Table S2.docx]

**Supplementary Table S2**: The oligonucleotide primer sequences used in this study

| **Target gene** | **Primer Name** | **Primer Sequence (5′-3′)** | **Amplicon**  **Size (bp)** | **Reference** |
| --- | --- | --- | --- | --- |
| *usp*A | *usp*A Up | CCGATACGCTGCCAATCAGT | 884 | [30] |
|  | *usp*A Down | ACGCAGACCGTAGGCCAGAT |  |  |
| *uid*A | *uid*A Up | TATGGAATTTCGCCGATTTT | 164 |  |
|  | *uid*A Down | TGTTTGCCTCCCTGCTGCGG |  |  |
| *mcr*-1 | *mcr1*_320bp_fw | AGTCCGTTTGTTCTTGTGGC | 320 | [37] |
|  | *mcr1_320bp_rev* | AGATCCTTGGTCTCGGCTTG |  |  |
| *mcr*-2 | *mcr2_700bp_fw* | CAAGTGTGTTGGTCGCAGTT | 715 |  |
|  | *mcr2*_700bp_rev | TCTAGCCCGACAAGCATACC |  |  |
| *mcr*-3 | *mcr3*_900bp_fw | AAATAAAAATTGTTCCGCTTATG | 929 |  |
|  | *mcr3*_900bp_rev | AATGGAGATCCCCGTTTTT |  |  |
| *mcr*-4 | *mcr4*_1100bp_fw | TCACTTTCATCACTGCGTTG | 1,116 |  |
|  | *mcr4*_1100bp_rev | TTGGTCCATGACTACCAATG |  |  |
| *mcr*-5 | *MCR5*_fw | ATGCGGTTGTCTGCATTTATC | 1,644 |  |
|  | *MCR5*_rev | TCATTGTGGTTGTCCTTTTCTG |  |  |
